# Selected acute phase protein in the diagnosis of late allergic response and the development of tolerance in food allergy

**DOI:** 10.1186/2045-7022-3-S3-P95

**Published:** 2013-07-25

**Authors:** EM Springer, M Sobieska

**Affiliations:** 1 Center of Diagnostics and Treatment of Allergy, SNZOZ Alergologia Plus, Poznań, Poland; 2 Chair and Clinic for Physiotherapy, Rheumatology and Rehabilitation, University of Medical Sciences, Poznań, Poland

## Background

Food allergy as an immunological disorder leads to specific local and general inflammation. There are no confident tests to confirm the diagnose of late reaction of food allergy or developing tolerance. Acute phase proteins (app) are markers of inflammation even localized or of very low intensity and their modulator effect on the immune reaction was proved in many studies. Since anti-chymotripsine (ACT) influences processes of induction, cells’ migration and effector mechanisms of allergic reaction we studied the usefulness of estimation of ACT level and its glycosylation profile (W1,W2, W3, W4 glycoforms) in diagnostic process of food allergy/ tolerance in children.

## Methods

The investigated group consisted of 40 children aged from 2 to 13 years with food allergy to cow’s milk. Two control groups, sex and age matched, included: 21 non-allergic children with other inflammation process **(IC)** and 13 healthy children **(HC).** With all children open food challenge with cow’s milk was performed. Upon its result an investigated group was divided into 3 groups: immediately reacting (**IR=16**), late reacting (**LaR=16)** and secondary tolerating (**T=14)** the allergen. Blood samples were taken before the challenge (**0**), 1 hour after (**1**) and 3 hours after (**2**). The level of ACT and its glycophorms were estimated by appropriate methods and analyzed statistically.

## Results

The basal level (**0**) of ACT was statistically significantly lower with different glycosylation profile in LaR group in relation to T, IC, HC(p<0.001) and IR (p<0.01) groups. Diagnostic values of these estimations for prediction a late allergic reaction reached likelihood ratio (LR) 5.0-14.3, with high sensitivity (80-100%), specificity (67-100%), predictive value positive (89-100%) and negative (86-100%). Furthermore high percentage of W4 glycoform seen in the group T had a high diagnostic value for differentiation with the LaR and HC groups. After food challenge significant changes in ACT level and its glycosylation profile were seen only in children not reacting to food: T, HC and IC (LR 3-6,4). Lack of these changes in sample 1 and 2 marks out mainly the IR group.

## Conclusion

These results show that estimations of ACT’s level and its glycosylation profile offer valuable contribution for diagnosis of food allergy and tolerance especially in case of late allergic reaction.

## Disclosure of interest

None declared.

